# Hospital Clinicians’ Knowledge of and Opportunity and Motivation for Prescribing Short Antibiotic Courses for Common Infections

**DOI:** 10.3390/pharmacy13020038

**Published:** 2025-03-01

**Authors:** Michael Wilcock, Dan Hearsey, Mandy Slatter, Neil Powell

**Affiliations:** 1 Pharmacy Department, Royal Cornwall Hospitals NHS Trust, Truro TR1 3LJ, UK; daniel.hearsey1@nhs.net (D.H.); neil.powell2@nhs.net (N.P.); 2 Pharmacy Department, Royal United Hospitals NHS Foundation Trust, Bath BA1 3NG, UK; mandy.slatter@nhs.net

**Keywords:** antibiotics, antimicrobial resistance, antimicrobial stewardship, guidelines, medical staff

## Abstract

Short-course antibiotic therapies for common infections treated in hospital are supported by national guidelines. Hospital clinicians’ knowledge of the course length recommendations for the management of common infections has not been fully explored. This study aims to assess doctors’ knowledge of and explores their opportunity and motivation for prescribing short-course therapy. A survey was emailed to all prescribers working in adult medical specialties in two hospitals in England. The survey responses from both hospitals were pooled before analysis. One hundred and sixty-five responses were provided. Knowledge of the recommended short course lengths was high overall, except for severe community-acquired/hospital-acquired pneumonia (CAP/HAP), with only 44% of respondents opting for shorter-course therapy. The majority did not believe longer courses were more effective than shorter courses. We identified a gap in prescriber knowledge for appropriate antibiotic course lengths for severe CAP/HAP. Addressing this gap may contribute to antimicrobial stewardship efforts to reduce course lengths in line with national guidelines.

## 1. Introduction

Antibiotic use drives antimicrobial resistance (AMR), with longer antibiotic durations increasing the risk of AMR and patient harm due to side effects. Compared to traditional longer antibiotic courses, short-course therapy has been shown to have comparable rates of infection cure, relapse and mortality [1,2,3]. Protracted course lengths are associated with an increased risk of antibiotic-associated harm [4]. Short-course therapies for common infections treated in hospital are supported by national guidelines [5], and highlighting the duration of therapy as part of clinician education programmes (both as a concept and in discussions of treating specific clinical scenarios) is a recommended antimicrobial stewardship (AMS) intervention [6]. It has been shown that compliance with these guidelines on short antibiotic course lengths for common infections had potential to reduce antibiotic use in medical specialties by 12.4% and reduce overall hospital usage by 3.6% [7]. Whilst subsequent audits by the same authors found that approximately half the patients treated for common medical infections received short-course therapy and half received longer courses (measured in days of therapy), and despite several audit feedback cycles, the ratio of short courses to longer courses remains at about 50% [8]. One review of behavioural drivers that influenced (prolonged) duration of antibiotic therapy in the hospital setting described a wide range of determinants, including healthcare professionals’ knowledge or expertise about the targeted condition/recommendation and the skills needed to adhere to the recommended practice [9].

The aim of this study was to assess clinicians’ knowledge of the current recommended antibiotic course lengths, and awareness of the evidence that supports these recommendations, for community-acquired pneumonia (CAP), hospital-acquired pneumonia (HAP), infective exacerbation of chronic obstructive pulmonary disease (IECOPD), cellulitis and pyelonephritis. An additional aim was to explore their motivations, and the opportunity for prescribing short antibiotic course lengths, to further understand the barriers to optimising short-course therapy.

## 2. Materials and Methods

An online cross-sectional survey study was conducted amongst prescribers working in adult medical specialties in two healthcare Trusts in the SW of England. There was no requirement for formal ethics committee approval under the Health Research Authority criteria. Completely anonymous survey procedures were used, and no identifying data were collected.

A Google Forms open survey, comprising 30 questions displayed on one page, was developed using the COM-B behaviour change model [10], to explore the barriers to and enablers of prescribing short antibiotic course lengths. The questions were developed by a member of the study team and piloted within the study team. The questions were not set as mandatory to complete, and once answers were submitted, the respondents could not change their responses; any incomplete responses or duplicated responders were excluded. The link to the survey was emailed to all prescribers working in adult medical specialties in two healthcare Trusts in the SW of England. The respondents were able to access the link voluntarily. Reminders were sent five times over a 6-week period between 2 November 2023 and 10 February 2024. All data were submitted anonymously, and anonymised consent was obtained for participation and publication.

The Royal Cornwall Hospitals NHS Trust (RCHT) is a 760-bed district general hospital serving a population of 450,000 people in a largely rural part of the UK. Royal United Hospitals NHS Foundation Trust (RUHB) is a 732-bed district general hospital serving a population of approximately 500,000 residents in a mixture of urban and rural areas in the SW of England. Both hospitals have an electronic prescribing system and both hospitals have antimicrobial guidelines in place.

The survey responses from both hospitals were pooled and analysed to determine clinicians’ knowledge of recommended course lengths for common infections treated in hospital, and the influences on clinicians’ opportunity and motivation for prescribing shorter or longer course lengths. We grouped the responses in a 2 × 2 table and used a Chi Squared (X^2^) test to determine whether the responses from each hospital differed.

## 3. Results

A total of 171 responses were completed: 84/367 (23%) from RCHT and 87/363 (24%) from RUHB; the overall response rate was 23% (Table 1). A total of 124 responders were medical doctors (75%) compared with 31 from surgical specialities (19%); 10 responders (6%) did not state their speciality. Six respondents’ data were subsequently excluded due to incomplete data.

Knowledge about the duration of short-course therapy was highest for non-severe CAP/HAP, with 88% of respondents correctly choosing 5 days, and lowest for severe CAP/HAP, with only 44% choosing 5 days (Table 2).

Overall, across the various infections, the majority of respondents (81%) were either unaware or not sure of the evidence for short-course therapy (Table 3). Awareness of the evidence for short-course therapy was highest for CAP and HAP (32% of respondents reporting awareness of the evidence) and lowest for cellulitis (9% aware of the evidence).

Most respondents (81%) said course length recommendations were clearly indicated in the hospital’s guidelines. The majority (89%) responded they were able to prescribe the courses they believed to be adequate for their patients, with only 11% of respondents indicating that they receive pushback from colleagues when they try to prescribe short course lengths they believe to be most appropriate. Seventy-seven percent of respondents agreed that the electronic-prescribing system facilitated prescribing appropriate course lengths for patients.

The majority of respondents felt confident prescribing short antimicrobial courses for common infections (80%). Most respondents (76%) stated that they were convinced by the evidence for short-course therapy; 6% were not convinced by the evidence; and 12% were unaware of the evidence. The majority of respondents (81%) stated that they did not prescribe longer course lengths to protect themselves as prescribers (that is, being in a position to retrospectively defend their decision if ever asked), and the majority (87%) did not believe that longer course lengths were better than shorter course lengths at treating infections. Nearly all (95%) of the respondents agreed that long antibiotic course lengths put patients at greater risk of side effects, and the majority of respondents (84%) did not believe that shorter course lengths put patients at greater risk of future antimicrobial resistant infections.

Sixty-six percent of all respondents stated that slow-to-resolve symptoms prevented them from prescribing short antibiotic courses, and 82% of respondents stated that diagnostic uncertainty contributed to their prescribing of longer antibiotic courses. Nearly all (94%) agreed that an evidence-based biomarker would help them to prescribe short course lengths.

Ninety-two percent indicated that antimicrobial guidelines approved by hospital infection specialists and hospital clinical management motivated them to prescribe short-course therapy. The majority of respondents (87%) indicated that ward pharmacist challenge of protracted course lengths would motivate them to prescribe short-course therapy, as would regular audits and feedback (84% agreed). Eighty-two percent agreed that a local champion advocating short-course therapy would motivate them to prescribe short-course therapy.

There was no statistically significant difference between the two hospitals in terms of knowledge of short courses for the common infections, except for pyelonephritis (Table 4). The recommended course lengths for pyelonephritis, although both comply with national guideline recommendations, differed between the two hospitals (7 days for RCHT, 7–10 days for RUHB), which may explain this result.

There were no differences in responses from the two hospitals to questions that explored opportunity. As regards motivation questions, there was one significant difference in the response to whether respondents thought approved guidelines that advocate short-course therapy motivated them to prescribe short-course therapy, with 94% from RCHT and 100% from RUHB answering yes (*p* = 0.02).

## 4. Discussion

We identified a gap in knowledge about appropriate course lengths for severe CAP and HAP, with the majority opting for protracted course lengths for this group of patients. Knowledge of the recommended short antimicrobial course lengths was high for the remaining common infections included in this survey. It is perhaps not surprising that prescribers in Canada, at least in 2019, were generally unaware that data exist to support shorter courses of antibiotic treatment [11]. We did not identify any significant opportunity barriers to prescribing short-course therapy. Confidence with prescribing short-course therapy was high (though they did not necessarily know the evidence base) and most were aware that shorter courses were as effective as longer courses and that longer courses increased the risk of side effects and increased the AMR risk. Factors that contribute to protracted courses lengths are diagnostic uncertainty and slow-to-improve clinical signs of infection, with only a minority prescribing longer courses to protect themselves as prescribers. Others have found across hospitals in Scotland that longer duration of antibiotic therapy was observed among older patients, possibly reflecting greater complexity or clinical uncertainty [12].

A relatively large sample of respondents were approached from two different hospitals within SW England; low response rates from both hospitals were observed, which may affect the generalisability and validity of our results and threaten the validity of our conclusions, especially given potential response bias. This survey asked clinicians to self-report their knowledge of the evidence base; therefore, our findings may be subject to self-report bias. Furthermore, this knowledge was not assessed within the survey. The survey allowed respondents to indicate free-text answers, so the homogeneity of the responses is not absolute, with some responses classified as ‘Other’, although these numbers were small. We were unable to analyse the answers of the respondents to ascertain if there were any differences in knowledge/confidence/motivation based on the “prescribing etiquette” of the job role or clinicians’ seniority, or years of practice that affected their decision-making regarding prescribing short courses [13]. It has been shown that junior doctors are often reluctant to alter initial prescriptions from senior doctors [9]; hence, this is an opportunity to further explore if the seniority or experience of a prescriber has implications for knowledge of, and belief in following, guideline-recommended durations of antibiotic therapy.

The factors that the majority of respondents acknowledged as motivation to prescribe short courses have been described elsewhere. An evidenced-based biomarker with high sensitivity and specificity for infection resolution is critical to the goals of individualised patient care and effective AMS [14], whilst infection specialist- and hospital-approved antimicrobial guidelines that advocate short-course therapy, ward pharmacist challenge of protracted course lengths and audit feedback are also key to AMS programmes [15].

Various studies have shown excessive antibiotic duration in hospitalised patients [16]. For instance, Vaughn et al. identified that two-thirds of hospitalised patients with pneumonia were prescribed excess course lengths in a US study [4]. The barriers and enablers to short antibiotic course lengths were explored in long-term care facilities in Canada and, similar to our finding, found that the speed of recovery influences the course lengths prescribed [11]. Similarly, guidelines and pharmacists were cited as important resources when determining appropriate course lengths, and clinicians identified up-to-date evidence of shorter durations as a key factor in changing their prescribing habits. Contrary to our findings, Langford et al. found that clinicians, albeit not from a hospital setting, were concerned with lower efficacy of shorter courses and concerned about potential for increased rates of resistance with shorter courses [11], a once common belief that is now outdated [17].

As reported elsewhere [18,19], we will introduce quality improvement initiatives to provide focused education on short courses in severe CAP/HAP and utilise the opportunity for pharmacists to check and query inappropriate course lengths at the point of dispensing discharge prescriptions. The role of external team members such as pharmacists has been reported to increase clinicians’ confidence about stopping potentially unnecessary antibiotics [9], and indeed, hospital pharmacists have been shown to have a critical role in antimicrobial stewardship activities [20].

We recognise that our survey did not set out to address patients’ acceptance and understanding of short-course antibiotics, which may impact the effectiveness of implementing such courses in real clinical settings.

## 5. Conclusions

We identified a gap in prescriber knowledge for appropriate antibiotic course lengths for severe CAP/HAP. Addressing this knowledge gap may contribute to AMS efforts to reduce course lengths in line with national guidelines. Hence, as part of recommended quality improvement initiatives, we intend to deliver target education on short courses in severe CAP/HAP plus another round of audit feedback augmented by pharmacist challenge of course lengths on discharge prescriptions.

## Figures and Tables

**Table 1:** Role of respondents.

Breakdown by Role	RCHT	RUHB
Advanced Clinical Practitioner	0	1
Associate Specialist	2	4
Clinical Fellow/F3	0	2
Consultant	20	38
Core Trainee/Registrar	10	1
Foundation Year Doctor	27	10
Non-medical Prescriber	3	11
Registrar	7	8
Surgical House Office not in training	0	1
Specialist Trainee/Registrar	11	5
Trust Doctor/grade	0	2
General Practice Speciality Trainee 2	1	0
Specialty Doctor	1	0
Excluded (role not provided)	2	4
Total responses	84	87
Specialty	Total	
Medical	124	
Surgical	31	
Not specified	10	
Excluded	6	
Total	171	

**Table 2:** Proportion of appropriate responses to questions about recommended course lengths for common infections surveyed. N = 165. Also shown are recommended antibiotic course lengths in participant hospitals’ antibiotic guidelines (RCHT hospital, RUHB hospital).

Infection	Respondents Stating Course Lengths in Line with Recommendations as Per Local Guidelines (%)	Respondents Stating Protracted Courses (%)	Respondents Stating Other Course Lengths (%)	Recommended Course Length at Each Trust RCHT RUHB	
Non-severe CAP/HAP	145 (88%)	8 (5%)	12 (7%)	5 days	5 days
Severe CAP/HAP	72 (44%)	90 (55%)	3 (2%)	5 days	5 days
Cellulitis	141 (85%)	17 (10%)	7 (4%)	5 days	5 days
Pyelonephritis	108 (65%)	39 (24%)	18 (11%)	7 days	7–10 days
IECOPD	130 (79%)	27 (16%)	8 (5%)	5 days	5 days
Total (%)	596 (72%)	181 (22%)	48 (6%)		

CAP; community-acquired pneumonia, HAP; hospital-acquired pneumonia, IECOPD; infective exacerbation of chronic obstructive pulmonary disease.

**Table 3:** Proportion of appropriate responses to questions about knowledge of evidence for short courses. N = 165.

Knowledge of Published Evidence to Support Short-Course Therapy	Yes	No	Not Sure	Blank
CAP and HAP	54 (32%)	82 (50%)	29 (18%)	0
IECOPD	35 (21%)	91 (56%)	37 (22%)	2 (1%)
Cellulitis	15 (9%)	128 (76%)	21 (13%)	1 (1%)
Pyelonephritis	18 (11%)	122 (74%)	25 (15%)	0
Overall	122 (18%)	423 (64%)	112 (17%)	3 (1%)

**Table 4:** Respondents’ knowledge of recommended short course length (RCHT hospital vs. RUHB hospital).

Indication	Trust	Recommended Course Length	Protracted and Other Course Lengths	Chi Squared Value	*p* Value
Non-severe CAP and HAP	RCHT	69	13	1.435	0.231
	RUHB	75	8		
Severe CAP and HAP	RCHT	38	44	0.485	0.4862
	RUHB	34	49		
IECOPD	RCHT	67	15	0.832	0.3617
	RUHB	63	20		
Cellulitis	RCHT	70	12	0	1
	RUHB	71	12		
Pyelonephritis	RCHT	47	35	4.775	0.0289
	RUHB	61	22		

## Data Availability

The data are available on request.
